# Tetrandrine alleviates oxaliplatin-induced mechanical allodynia via modulation of inflammation-related genes

**DOI:** 10.3389/fnmol.2024.1333842

**Published:** 2024-02-14

**Authors:** Zhi-Ling Zhang, Zi-Yang Wu, Feng-Yu Liu, Hang-YuChen, Suo-Di Zhai

**Affiliations:** ^1^Department of Pharmacy, Peking University Third Hospital, Beijing, China; ^2^Key Laboratory for Neuroscience, Neuroscience Research Institute and Department of Neurobiology, School of Basic Medical Sciences, Ministry of Education/National Health Commission, Peking University, Beijing, China

**Keywords:** tetrandrine, oxaliplatin, mechanical allodynia, inflammation, RNA-Seq, molecular docking

## Abstract

Oxaliplatin, a platinum-based chemotherapy drug, causes neuropathic pain, yet effective pharmacological treatments are lacking. Previously, we showed that tetrandrine (TET), with anti-inflammatory properties, reduces mechanical allodynia in nerve-injured mice. This study explores the effect of TET on oxaliplatin-induced mechanical allodynia and gene changes in mice. Male C57BL/6J mice received oxaliplatin intraperitoneally to induce mechanical allodynia. Post-treatment with TET or vehicle, the mechanical withdrawal threshold (WMT) was assessed using von Frey filaments. TET alleviated oxaliplatin-induced mechanical allodynia. RNA sequencing identified 365 differentially expressed genes (DEGs) in the Control vs. Oxaliplatin group and 229 DEGs in the Oxaliplatin vs. TET group. Pearson correlation analysis of co-regulated DEGs and inflammation-related genes (IRGs) revealed 104 co-regulated inflammation-related genes (Co-IRGs) (|cor| > 0.8, *P* < 0.01). The top 30 genes in the PPI network were identified. Arg2, Cxcl12, H_2_-Q6, Kdr, and Nfkbia were highlighted based on ROC analysis. Subsequently, Arg2, Cxcl12, Kdr, and Nfkbia were further verified by qRCR. Immune infiltration analysis indicated increased follicular CD4 T cell infiltration in oxaliplatin-treated mice, reduced by TET. Molecular docking showed strong binding affinity between TET and proteins encoded by Arg2, Cxcl12, Kdr, and Nfkbia. In summary, TET may alleviate oxaliplatin-induced peripheral neuropathy in clinical conditions.

## Highlights

• Tetrandrine alleviated oxaliplatin-induced mechanical allodynia.
• Arg2, Cxcl12, H_2_-Q6, Kdr, and Nfkbia in the spinal cord were identified as inflammation-related genes in OINP and anti-OINP effect of tetrandrine.
• OINP and the anti-nociceptive effect of tetrandrine may be related to decreasing of follicular CD4 T cells.

## 1 Introduction

Oxaliplatin is a critical component in the treatment of colorectal cancer, and is also widely employed to combat various malignant tumors, including those found in the liver, stomach, esophagus, and pancreas (Zhang et al., 2022). However, 85–96% of patients experience acute oxaliplatin-induced neuropathy pain (OINP), while chronic OIPN commonly occurs in 40–93% of patients receiving oxaliplatin (Brozou et al., 2018). Symptoms of acute OINP are mainly manifested as sensitivity to touching cold objects, and for chronic OINP, pricking pain is the most severe symptom, which is not related to cold stimulation. Acute OINP often weakens between treatment cycles, but chronic OINP may persist for a long period of time even after the end of chemotherapy, with both severity and duration increasing as the dose of oxaliplatin accumulates (Sałat, 2020). This has led some patients to adjust their dosage or even interrupt treatment, affecting their treatment and quality of life (Kang et al., 2021). Studies have revealed that inflammation and immune system activity are central to chemotherapy-induced neuropathic pain (CINP), and CINP may be alleviated by inhibiting pro-inflammatory responses or augmenting anti-inflammatory immune cells and cytokines (Xu et al., 2018; Dong et al., 2022a,b; Lee et al., 2022).

The plant *Stephania tetrandra* S. Moore is commonly used in traditional Chinese medicine for its anti-inflammatory, diuretic, and antirheumatic properties (Jiang et al., 2020). Tetrandrine (TET) is a bisbenzylisoquinoline alkaloid extracted from the root of *Stephania tetrandra* S. Moore. Its tablet formulation has been approved in China for the treatment of neuropathic pain (NP), rheumatoid arthritis, and other diseases. Its anti-inflammatory and antinociceptive effects have been reported. Zhao et al. (2014) reported that TET alleviates LPS-induced abnormal pain by inhibiting the production of prostaglandin 2 (PGE2) and cyclooxygenase 2 (COX-2) through the IKKβ/IκB/NF-κB pathway (Qin et al., 2018). TET also inhibited mechanical allodynia and interleukin 1β (IL-1β) and tumor necrosis factor alpha (TNF-α) of spared nerve injury induced NP mice (Zhang et al., 2023). However, it is currently unclear whether the anti-inflammatory effects of TET are protective in OINP. Therefore, we investigated the effect of TET on oxaliplatin-induced mechanical allodynia in mice and further studied the potential inflammation mechanism of TET’s action.

We applied oxaliplatin to induce mechanical allodynia to study the antinociceptive effect of TET. Subsequently, we extracted spinal cord (SC) tissues for RNA sequencing (RNA-Seq), which is a commonly used high-throughput transcriptomic technique to explore the potential molecular mechanisms and therapeutic targets of NP (Perkins et al., 2014; Du et al., 2018). Using the Illumina Hiseq 4000 platform, we investigated differentially expressed genes (DEGs). Furthermore, we carried out various bioinformatics analyses, such as Pearson correlation analysis, immune infiltration analysis, and molecular docking to reveal the potential anti-inflammatory mechanism of TET.

## 2 Materials and methods

### 2.1 Animals

All experimental procedures were approved by the Peking University Animal Use Committee (LA2021367) and complied with the National Institutes of Health Guide for the Care and Use of Laboratory Animals. Male C57BL/6J mice, weighing 20–22 g, were purchased from Beijing Vital River Laboratory Animal Technology Co., Ltd. Mice were given at least 7 days to adapt to the feeding environment before the experiments. Mice were randomly assigned and housed in a controlled environment (12-h light/dark cycle, temperature controlled).

### 2.2 Reagents

Tetrandrine (PubChem CID: 73078, C38H42N2O6) was provided by Zhejiang Kangenbei Medicine (Jinhua, Zhejiang, China), with a purity of 99.2%, and was prepared to the desired concentration using 0.5% sodium carboxymethyl cellulose before use. Oxaliplatin was acquired from TargetMol (Target Molecule Corp, Boston, MA, USA.), with a purity of 99.63%, and was diluted to the required concentration using sterile 5% glucose injection solution before application. Pregabalin with a purity of 99% was purchased from Energy Chemical (Shanghai, China) and dissolved in 0.9% sterile saline prior to use to the desired concentration.

### 2.3 Experimental protocols

**FIGURE 1:**
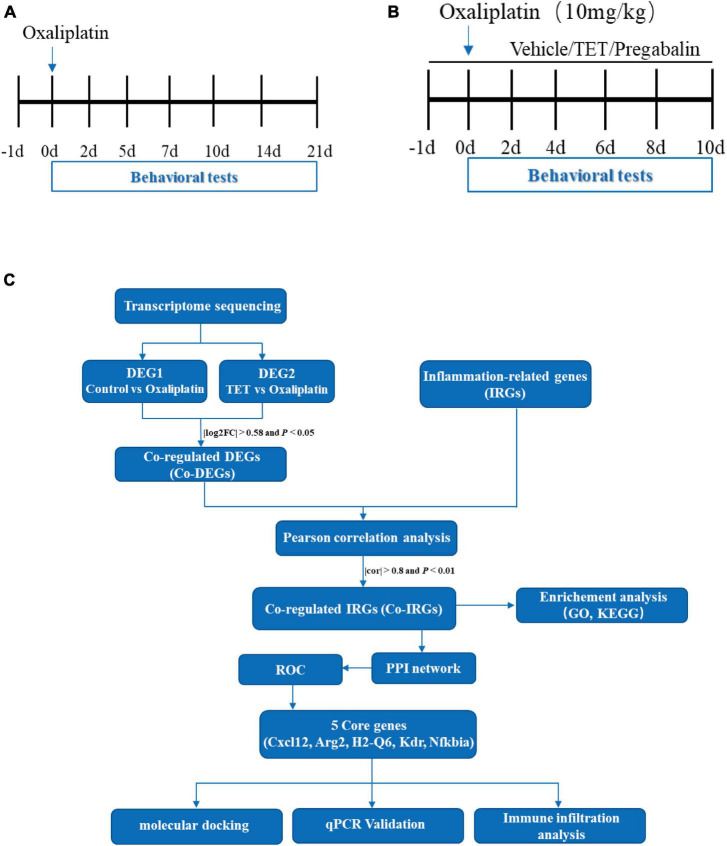
Comprehensive flow chart illustration of the experimental and data processing procedures. **(A)** Experimental design for model conduction: on day 0, a single intraperitoneal injection of oxaliplatin is administered, while the control group receives an equivalent volume of 5% glucose solution. Behavioral tests are conducted on days -1, 2, 5, 7, 10, 14, and 21. **(B)** Experimental design for testing tetrandrine (TET) in oxaliplatin-induced neuropathy Pain (OINP) mice: administration of TET or pregabalin via oral gavage begins one day prior to the oxaliplatin injection. Behavioral tests are performed every other day. **(C)** Data processing workflow in the study: following the completion of behavioral assessments, spinal cord tissues are collected for transcriptomic sequencing and qPCR validation. Bioinformatics analysis is then conducted as illustrated.

Induction of OINP (Figure 1A): mice were randomly divided into three groups, each with *n* = 6: Vehicle group, Oxaliplatin 6 mg/kg group (Kim et al., 2017), and Oxaliplatin 10 mg/kg group (Huynh et al., 2022). A single intraperitoneal administration at a volume of 10 mL/kg was given, and animals in the Vehicle group received an equal volume of sterile 5% glucose injection solution.

Tetrandrine’s anti-OINP effect (Figure 1B): mice were randomly divided into 4 groups, with *n* = 6 in each group: Control group, Oxaliplatin group, TET group, and Pregabalin group. Mice in the Control group received oral gavage of 0.5% carboxymethylcellulose sodium from day -1 to day 10 and a single intraperitoneal injection of sterile 5% glucose on day 0. Mice in the Oxaliplatin group received gastric gavage of 0.5% carboxymethylcellulose sodium from day -1 to day 10 and a single intraperitoneal injection of 10 mg/kg oxaliplatin on day 0. Mice in the TET group received gastric gavage of 45 mg/kg TET from day -1 to day 10 and a single intraperitoneal injection of 10 mg/kg oxaliplatin on day 0. Mice in the Pregabalin group received gastric gavage of 45 mg/kg Pregabalin from day -1 to day 10 and a single intraperitoneal injection of 10 mg/kg oxaliplatin on day 0.

### 2.4 Mechanical allodynia

Mechanical allodynia was evaluated by measuring the mechanical withdrawal threshold (MWT) by employing a range of von Frey filaments (0.008–2.0 g) applied to the central region of the mouse’s foot sole. Prior to the experiment, mice were placed in plastic boxes on metal grids four times, for 30 min each time, to adapt to the experimental environment. The up-down method was used to determine the MWT (Dixon, 1980). If a mouse exhibited withdrawal, trembling, or licking its hind paw within 5 s of vertical stimulation the central part of the foot sole (starting with a 0.6 g filament), it was considered a pain response, and a weaker filament was selected; otherwise, a stronger fiber filament was applied. After reaching the first crossover, four additional stimuli were applied, and the last six stimuli were used to calculate the 50% MWT (Dixon, 1980). All behavioral tests were conducted by investigators who were unaware of the experimental groups.

### 2.5 Rotarod test

Rotarods were utilized to evaluate the motor function of mice following TET administration. After gastric gavage of TET or vehicle, mice were positioned on a rotating rod, which compelled them to walk against its movement at a speed of 30 rpm. We recorded time it took for the mice to fall from the rod, with a maximum observation time of 180 s. Prior to the baseline assessment, the mice underwent four training sessions.

### 2.6 Tissue collection, cDNA library construction, and RNA-Seq

The RNA-Seq and bioinformatic analysis flow chart of this study is shown in Figure 1C. After the final MWT test, the lumbar enlargement tissues of the SC of mice from control, oxaliplatin, and TET group were isolated. The RNA isolation, cDNA library construction, sequencing was carried out according to the Guoke Biotechnology (Beijing, China). Briefly, total RNA was extracted using the HiPure Universal RNA Mini Kit (Guangzhou, China, Magen Biotechnology Co., Ltd) according to the manufacturer’s protocol. RNA concentration and purity were measured using NanoDrop 2000. RNA integrity was assessed using the RNA Nano 6000 Assay Kit on the Agilent Bioanalyzer 2100 system. An RNA library was constructed using NEBNextR UltraTM Directional RNA Library Prep Kit according to the manufacturer’s recommendations and library quality was assessed on the Agilent Bioanalyzer 2100 system. RNA-seq was performed on the NovaSeq 6000 platform.

### 2.7 RNA preparation and quantitative real-time RT-PCR (qRT-PCR)

Total RNA was extracted from the Spinal dorsal horn utilizing Trizol reagent (Sigma-Aldrich). From 1 μg of the total RNA, first-strand cDNA was produced using the HiScript III RT SuperMix for qPCR (Vazyme), following the protocol provided by the manufacturer. The qRT-PCR analysis was conducted with the Quantstudio 5 (Applied Biosystems) and Taq Pro Universal SYBR qPCR Master Mix (Vazyme). The specific primer sequences for Cxcl12 were AGAAAGCTTTAAACAAGGGGCG and AGAGGGAGGAGCGAGTTACA; for Kdr were TCCA CATGGGCGAATCACTC and GAGTGTGCCAGCCTACTACA; for Arg2 were TCCTTGCGTCCTGACGAGAT and CAAGCCA GCTTCTCGAATGG; for Nfkbia were GAGACCTGGCCTTCCT CAAC and CAAGACTGCTACACTGGCCA; and for GAPDH were AGGTCGGTGTGAACGGATTTG and TGTAGACCAT GTAGTTGAGGTCAA. The relative expression levels were determined using the 2-ΔΔCt method. Ct values of Cxcl12, Kdr, Arg2, or Nfkbia genes were normalized against the GAPDH gene Ct values to account for Cxcl12, Kdr, Arg2, or Nfkbia gene expression changes. These normalized ΔCt values were then compared to the control ΔCt values to derive the ΔΔCt values. Finally, the expression levels were expressed as the ratio of 2-ΔΔCt relative to the control.

### 2.8 RNA-Seq data processing

Raw data (raw reads) in the FASTQ format were initially processed using an internal Perl script to obtain clean data (clean reads). These clean reads were then mapped to the reference genome sequence. The HISAT2 software tool (v2.2.1) was used for this mapping (Kim et al., 2019). Reads that are either an exact match or have only a single mismatch were then further analyzed and annotated based on the following databases: NR (NCBI non-redundant prote in sequences database), Nt (NCBI non-redundant nucleotide sequences),^1^ Pfam (The database of Homologous protein family),^2^ COG (The database of Clusters of Protein homology),^3^ Swiss-Prot (A manually annotated non-redundant protein sequence database),^4^ KOG (The database of Clusters of protein homology),^5^ KEGG (The database of Kyoto Encyclopedia of Genes and Genomes),^6^ and GO (Gene Ontology database).^7^ The level of gene expression was estimated using Fragments Per Kilobase of Transcript Per Million Fragments Mapped (FPKM). Principal component analysis (PCA) was performed based on the FPKM of all genes. Differentially expressed genes (DEGs) were extracted based on RNA-seq counts (|log2FC| > 0.58 and *P* < 0.05) using the DEseq2 package (v1.28.1) (Love et al., 2014). Four samples were used in each group for RNA- sequencing assay.

### 2.9 Functional annotations of DEGs

Gene Ontology and KEGG analysis were performed using the clusterProfiler package (*P* < 0.05) (Wu et al., 2021). The top 10 most significantly enriched GO and KEGG terms were extracted.

### 2.10 Analysis of co-regulated inflammation-related genes (co-IRGs)

Inflammation-related genes were retrieved from inflammation-related pathways collected in the MSigDB database,^8^ with the gene set as c5.go.bp.v7.5.1.symbols. Co-regulated DEGs (Co-DEGs) were obtained by overlapping DEGs between the Control-Oxaliplatin groups and the Oxaliplatin-TET groups. Pearson correlation analysis was employed to investigate the relationship between Co-DEGs and IRGs. The genes |cor| > 0.8 and *P* < 0.01 were identified as Co-IRGs.

### 2.11 Protein-protein interaction (PPI) network construction

The PPI network was established based on Co-IRGs through the STRING database (Szklarczyk et al., 2021). The top 30 genes were mined using MCC and Degree algorithms in the CytoHubba plugin (Chin et al., 2014), and the intersection was taken to obtain candidate genes.

### 2.12 Receiver operating characteristic (ROC) curve

The ROC curve was drawn using the pROC package (Robin et al., 2011) and depends on the expression of candidate genes and specimen grouping information. Cross genes were obtained by overlapping area under the curve (AUC) values greater than 0.7 between the Control-Oxaliplatin group and the Oxaliplatin-TET group. Subsequently, the cross genes were ranked based on the AUC values of the Control-Oxaliplatin group, and the top 5 genes were selected for subsequent analysis.

### 2.13 Immune infiltration analysis

The proportion of immune cells in the control group, oxaliplatin group, and TET group specimens was calculated using the CIBERSORT package and the mouse characteristic matrix (Chen et al., 2017). The correlation between central genes and immune cells was estimated through Pearson correlation analysis.

### 2.14 Molecular docking

The 3D structure of TET (CID: 73078) in SDF format was extracted from the PubChem database.^9^ High-resolution 3D structures of target proteins were downloaded from the RCSB Protein Database.^10^ These include Nfkbia (PDB ID: 1IKN), Arg2 (PDB ID: 4HZE), Kdr (PDB ID: 1YWN), Cxcl12 (PDB ID: 4UAI). PyMOL (v2.3.0) (Seeliger and de Groot, 2010) software was used to remove the original ligands and water molecules in the protein molecules. AutoDocktools (v1.5.6)^11^ was used for protein and drug hydrogenation, charge calculation, charge assignment, specifying atom types, and saving them as pdbqt format. Then, POCASA (v1.1)^12^ was used to predict the protein binding sites, and docking with AutoDock Vina (v1.1.2) (Trott and Olson, 2010). The interaction mode analysis of docking results was achieved through PyMOL and Ligplot (v2.2.5).

### 2.15 Statistical analysis

For statistical analysis, GraphPadPrism software (Inc., La Jolla, CA, USA) was used. The results are presented as mean ± standard error of the mean (SEM). The cumulative nociception and antinociceptive effect during the entire observation period was determined as the AUC of the time course. One-way analysis of variance (ANOVA) and Newman-Keuls tests were used to evaluate AUC and qRT-PCR data. Two-way ANOVA and Bonferroni’s *post-hoc* tests were employed to compare mechanical pain thresholds at multiple time points. Statistical significance was set at *P* < 0.05.

## 3 Results

### 3.1 Oxaliplatin-induced mechanical allodynia and the antinociceptive effect of TET

**FIGURE 2:**
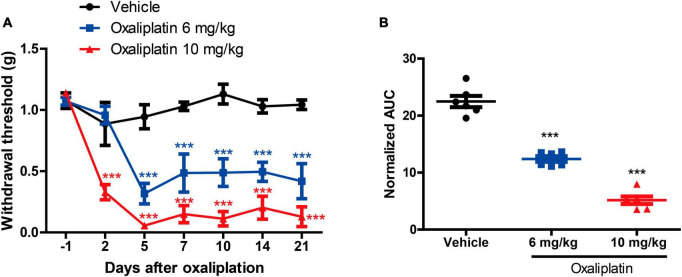
Establishment of an oxaliplatin-induced neuropathic pain mouse model. **(A)** The MWT data were examined 0, 2, 5, 7, 10, 14, and 21 days after oxaliplatin injection (i.p.), two-way ANOVA followed by Bonferroni’s *post-hoc* test. ***P < 0.001 compared to the vehicle at the same time point. **(B)** The area under the curve (AUC) of the data from graph B (2–21 days). One-way ANOVA followed by Newman-Keuls tests. ***P < 0.001 compared to the vehicle group. Data are presented as mean ± SEM, *n* = 6.

The results indicated that both 6 mg/kg and 10 mg/kg oxaliplatin caused mechanical hypersensitivity [two-way ANOVA, treatment: *F*_(2, 90)_ = 95.35, *P* < 0.0001; time: *F*_(6, 90)_ = 19.29, *P* < 0.0001; treatment × time: *F*_(12, 90)_ = 6.3, *P* < 0.0001] (Figure 2A). In comparison to the mice in the Vehicle group, those treated with 6 mg/kg oxaliplatin exhibited a decrease in MWT on days 5, 7, 10, 14, and 21, and mice treated with 10 mg/kg oxaliplatin demonstrated a decrease in MWT on days 2, 5, 7, 10, 14, and 21. Furthermore, the 10 mg/kg oxaliplatin treatment was found to reduce the MWT in mice more than the 6 mg/kg oxaliplatin treatment did. Similarly, the AUC values also indicated more obvious mechanical hypersensitivity at 10 mg/kg oxaliplatin [one-way ANOVA, *F*_(2, 15)_ = 137.5, *P* < 0.0001] (Figure 2B). However, compared to the Vehicle group, a single intraperitoneal injection of oxaliplatin (10 mg/kg) did not induce cold allodynia at -1, 1, 4, and 7 days (Supplementary Figure 1). Consequently, in subsequent experiments, the dose of 10 mg/kg oxaliplatin was selected for further study, with a primary focus on assessing mechanical allodynia as the main indicator.

**FIGURE 3:**
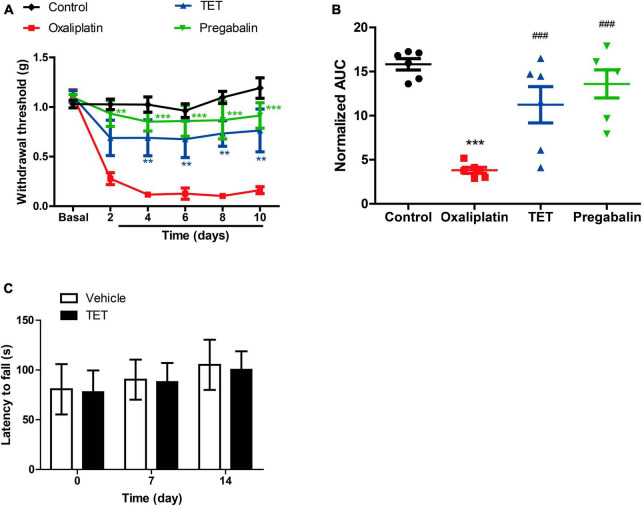
Tetrandrine (TET) alleviates oxaliplatin-induced mechanical allodynia. **(A)** The MWT data within each group were analyzed using two-way ANOVA followed by Bonferroni’s *post-hoc* test. **P < 0.01, ***P < 0.001 compared to Oxaliplatin at the same time point. **(B)** The area under the curve (AUC) of the data from graph B (2–10 days). ***P < 0.001 compared to Control group, ^###^*P* < 0.001 compared to the Oxaliplatin group. One-way ANOVA followed by Newman-Keuls tests. **(C)** Motor function was performed by rotarod test. Data are expressed as mean ± SEM, *n* = 6. TET and pregabalin use the dose of 45 mg/kg.

As Figure 3A shows, compared to the Oxaliplatin group, TET significantly increased the MWT on days 4, 6, 8, and 10, and pregabalin on days 2, 4, 6, 8, and 10 [two-way ANOVA, treatment: *F*_(3, 100)_ = 14.47, *P* < 0.0001; time: *F*_(5, 100)_ = 15.04, *P* < 0.0001; treatment × time: *F*_(15, 100)_ = 4.59, *P* < 0.0001]. The AUC results depicted in Figure 3B [one-way ANOVA, *F*_(3, 20)_ = 14.93, *P* < 0.0001] demonstrate that both TET and pregabalin ameliorated mechanical hypersensitivity in OINP mice. Interestingly, there was no statistical difference observed between these two groups.

To assess potential drug-induced effects on motor function, we compared the rotarod performance of mice treated with TET to those treated with a vehicle control. There were no significant differences between the groups, as shown in Figure 3C. This suggests that the antinociceptive effects of TET are not related to motor function.

### 3.2 Identification of DEGs in the control-oxaliplatin group and oxaliplatin-TET group

**FIGURE 4:**
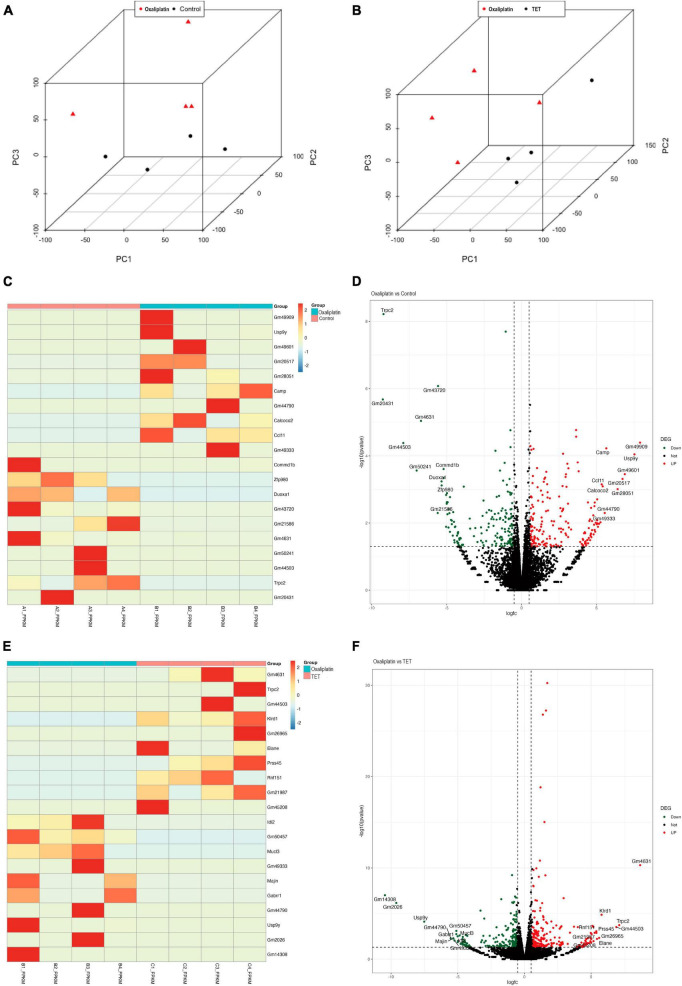
Identification of DEGs in Control-Oxaliplatin and Oxaliplatin-TET groups using DEseq2, with criteria of |log2FC| > 0.58 and *P* < 0.05. **(A)** PCA comparing Control and Oxaliplatin groups based on standardized gene expression. **(B)** PCA comparing Oxaliplatin and TET groups based on standardized gene expression. **(C)** Heatmap displaying top 10 DEGs between Control and Oxaliplatin groups. **(D)** Volcano plot illustrating DEGs distribution between Control and Oxaliplatin groups. **(E)** Heatmap displaying top 10 DEGs between Oxaliplatin and TET groups. **(F)** Volcano plot illustrating DEGs distribution between Oxaliplatin and TET groups. Red indicates upregulated genes, and green indicates downregulated genes.

To investigate molecular mechanisms underlying TET against OINP, we performed transcriptomic sequencing. The lumbar enlargement tissues of the SC from Control group, Oxaliplatin group and TET group were isolated (Figures 4A, B). The Control-Oxaliplatin group exhibited a total of 365 DEGs, including 194 upregulated and 171 downregulated genes (Figures 4C, D and Supplementary Table 1). The Oxaliplatin-TET group had a total of 423 DEGs, including 229 upregulated and 194 downregulated genes (Figures 4E, F and Supplementary Table 2).

### 3.3 GO and KEGG analysis

**FIGURE 5:**
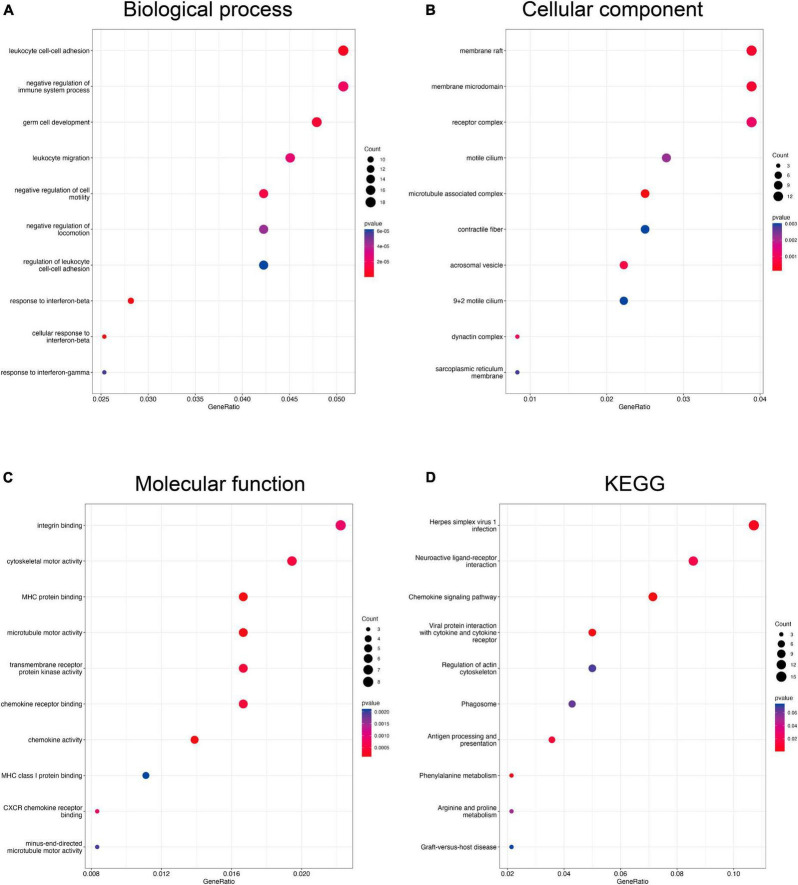
Functional enrichment analysis of DEGs compare the Oxaliplatin and TET groups using clusterProfiler. The bubble charts showed BP **(A)**, CC **(B)**, MF **(C)**, and KEGG **(D)**.

To understand the potential functions of DEGs, we performed GO and KEGG enrichment analysis. The GO analysis for DEGs of the Control-Oxaliplatin group showed that for biological processes (BP), these DEGs were related to homeostasis of the number of cells, negative regulation of cytokine production, and antimicrobial humoral response (Supplementary Figure 2). For cellular components (CC), these DEGs were associated with secretory granules, bicellular tight junctions, and tight junctions (Supplementary Figure 2). As for molecular functions (MF), these DEGs were related to G protein-coupled receptor binding, chemokine receptor binding, and antioxidant activity (Supplementary Figure 2). Meanwhile, KEGG results suggested that DEGs between the Control and Oxaliplatin groups were related to cytokine-cytokine receptor interaction, MAPK signaling pathway, and tight junctions (Supplementary Figure 2). GO results suggested that DEGs in the Oxaliplatin-TET group were related to leukocyte cell-cell adhesion, negative regulation of the immune system process, and germ cell development in terms of BP (Figure 5A). In terms of CC, they were related to membrane rafts, membrane microdomains, and receptor complexes (Figure 5B). In terms of MF, they were related to integrin binding, cytoskeletal motor activity, and MHC protein binding (Figure 5C). KEGG analysis results showed that DEGs between the Oxaliplatin and TET groups were related to herpes simplex virus 1 infection, neuroactive ligand-receptor interaction, and chemokine signaling pathway (Figure 5D).

### 3.4 Identification of co-IRGs and functional enrichment

**FIGURE 6:**
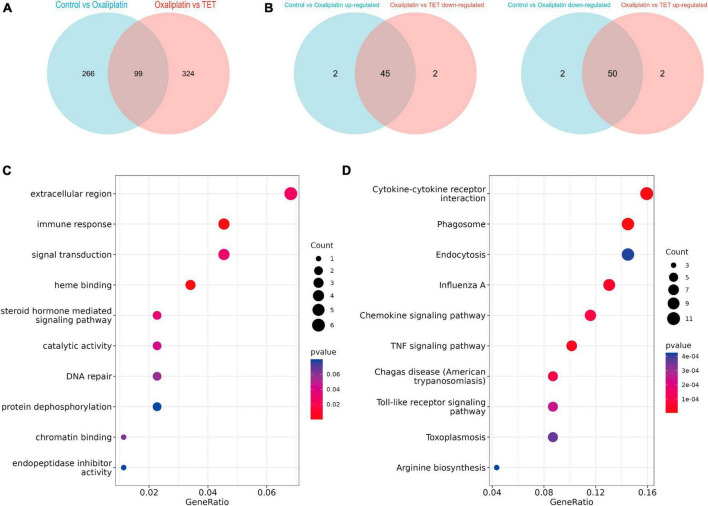
Identification and functional annotation of Co-regulated IRGs. **(A)** The Venn diagram displays 99 Co-regulated DEGs between the Control-Oxaliplatin and Oxaliplatin-TET groups. **(B)** The Venn diagrams indicate that the expression profiles of 95 Co-regulated DEGs were reversed following TET treatment. **(C)** A bubble chart shows the enriched GO terms for Co-regulated IRGs. **(D)** A bubble chart presents the enriched KEGG terms for Co-regulated IRGs.

To explore the anti-inflammatory mechanism of TET against OINP, IRGs were retrieved from inflammation-related pathways collected in the MSigDB database (Supplementary Table 3). Meanwhile, 99 Co-DEGs were obtained by overlapping the DEGs of the Control-Oxaliplatin group with those of the Oxaliplatin-TET group (Figure 6A and Supplementary Table 4). Among these, 45 DEGs upregulated in the Control-Oxaliplatin group were downregulated in the Oxaliplatin-TET group, while 50 DEGs downregulated in the Control-Oxaliplatin group were upregulated in the Oxaliplatin-TET group (Figure 6B). Then 104 Co-IRGs (|cor| > 0.8 and *P* < 0.01) were identified by conducting Pearson correlation analysis of Co-DEGs and IRGs (Supplementary Table 5). GO and KEGG analyses were used to explore the potential functions of Co-IRGs. GO results showed that Co-IRGs were related to the extracellular region, immune response, and signal transduction (Figure 6C). Moreover, KEGG analysis results indicated that Co-IRGs were related to cytokine-cytokine receptor interaction, TNF signaling pathway, and toll-like receptor signaling pathway (Figure 6D).

### 3.5 PPI network analysis

**FIGURE 7:**
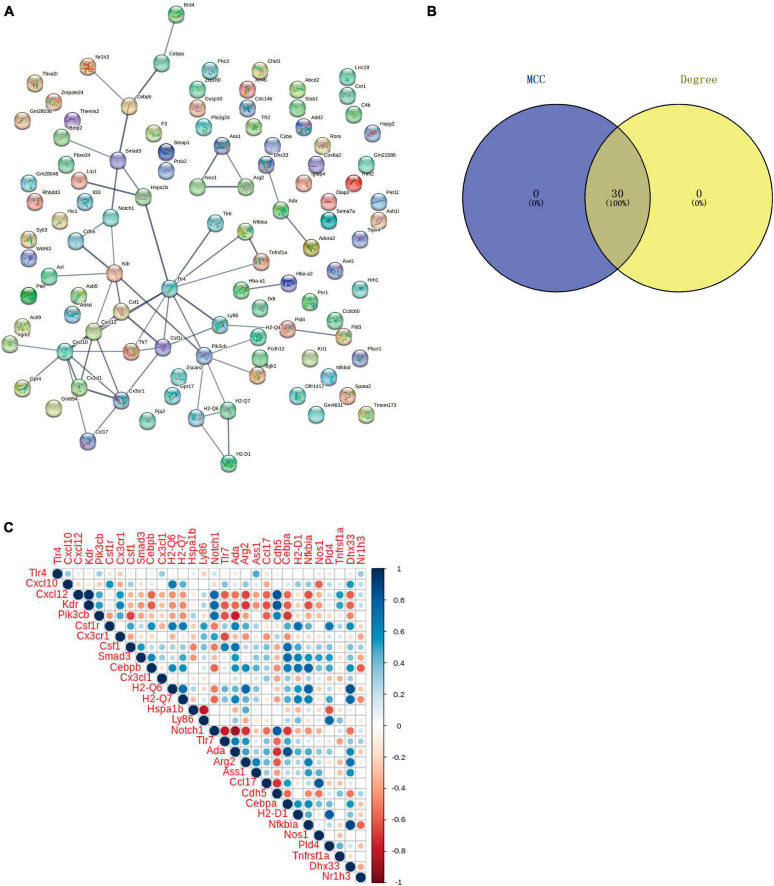
Protein-protein interaction (PPI) network and correlation analysis of co-regulated IRGs. **(A)** PPI network of Co-regulated IRGs established using the STRING database. **(B)** Venn diagram of candidate genes identified through MCC and Degree algorithms. **(C)** Correlation of the 30 candidate genes.

**FIGURE 8:**
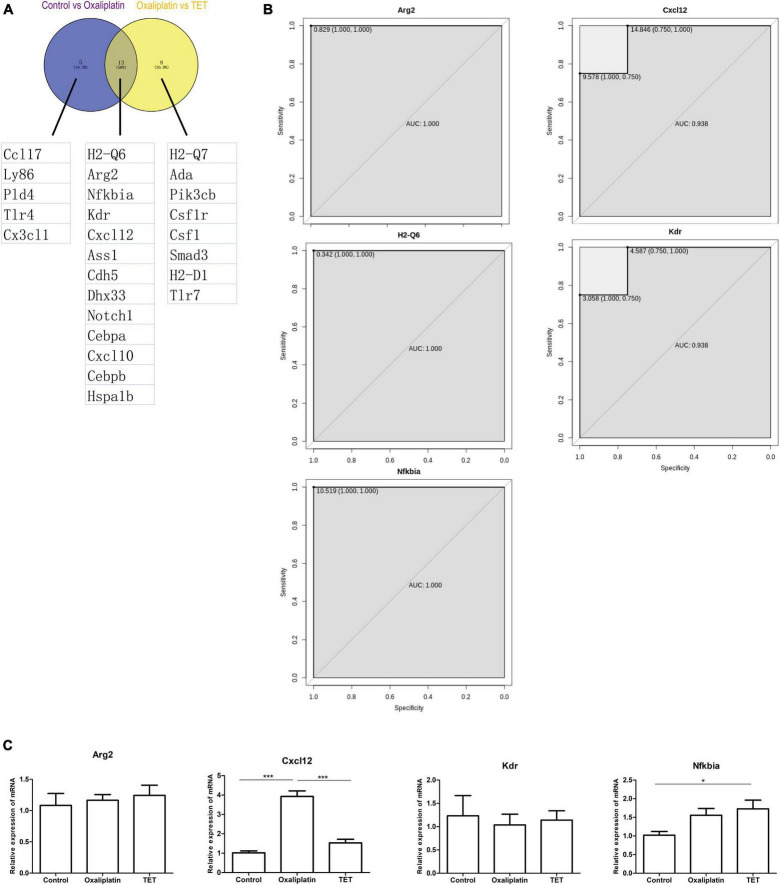
Receiver operating characteristic analysis. **(A)** Venn diagram of 13 genes with AUC values greater than 0.7 for both Control-Oxaliplatin and Oxaliplatin-TET groups. **(B)** ROC analysis of Arg2, Cxcl12, Kdr, H_2_-Q6, and Nfkbia in the Control-Oxaliplatin group with AUC values greater than 0.8. **(C)** Effects of TET on Arg2, Cxcl12, Kdr, and Nfkbia expressions in oxaliplatin induced NP. One-way ANOVA followed by Newman-Keuls tests. *P < 0.05, ***P < 0.001. Data are presented as mean ± SEM, *n* = 5.

Protein-protein interaction analysis of Co-IRGs was conducted (Figure 7A). Subsequently, MCC and Degree were employed to identify the top 30 genes in the PPI network (Figure 7B). Figure 7C showed the correlation of the 30 candidate genes. Next, those 30 genes were subjected to ROC analysis. As Figure 8A shows, the AUC values of 18 genes in the control-‘ group (Supplementary Table 6) and 21 genes in the Oxaliplatin-TET group (Supplementary Table 7) exceeded 0.7. According to the ranking of AUC values in the Control-Oxaliplatin group, five genes (H_2_-Q6, Arg2, Nfkbia, Kdr, and Cxcl12) were identified (Supplementary Table 8). Figure 8B and Supplementary Figure 3 show that the AUC values of these 5 genes in the Control-Oxaliplatin group and Oxaliplatin-TET group are both greater than 0.8. Compared with the Control group mice, Kdr and Cxcl12 were increased in the Oxaliplatin group mice, while H_2_-Q6 was decreased in the Oxaliplatin group mice. TET decreased the expression of Kdr and Cxcl12 but increased the expression of H_2_-Q6. Arg2 and Nfkbia were IRGs highly correlated with Co-DEGs. The qRT-PCR validation results, as shown in Figure 8C, reveal that TET significantly decreased the mRNA expression levels of Cxcl12, which were increased by oxaliplatin. Additionally, TET appeared to upregulate Nfkbia expression, with levels significantly higher than those in the control group. However, the expression levels of Arg2 and Kdr did not show significant differences among the groups.

### 3.6 Immune infiltration analysis

**FIGURE 9:**
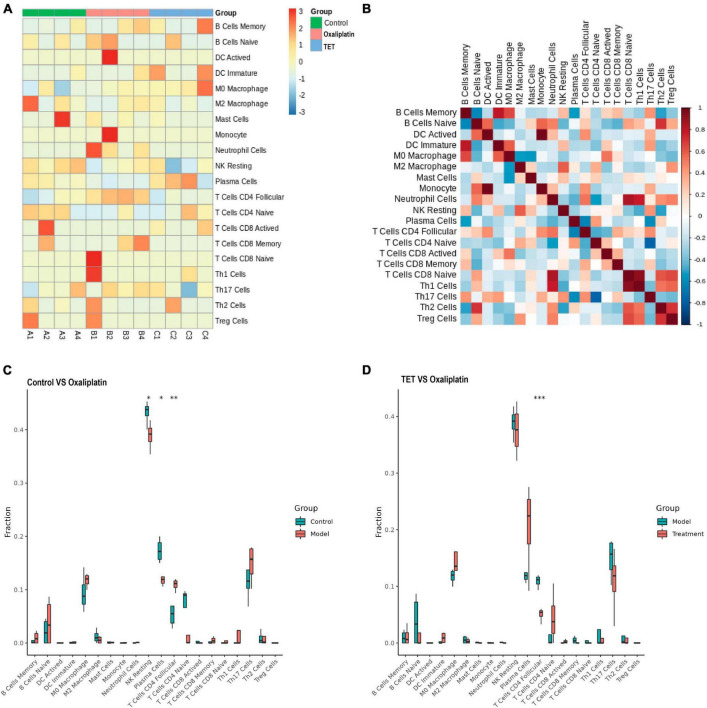
Immune infiltration analysis. **(A)** Heatmap displaying infiltration abundances of 20 immune cells across Control, Oxaliplatin, and TET groups. **(B)** Pearson correlation heatmap of 20 immune cells. **(C)** Boxplot comparing immune cell infiltration between Control and Oxaliplatin groups. **(D)** Boxplot comparing immune cell infiltration between Oxaliplatin and TET groups.

Due to the strong association between inflammation and the immune system, we carried out immune infiltration analysis. After removing unexpressed immune cells in mice, the abundance of 20 immune cells was shown in Figures 9A, B shows the correlation of the 20 immune cells. CIBERSORT analysis revealed that, compared with the control group, oxaliplatin decreased the infiltration of resting NK cells and plasma cells, but increased the infiltration of follicular CD4 T cells (Figure 9C). Conversely, TET decreased the infiltration of follicular CD4 T cells (Figure 9D). The correlation analysis demonstrated that follicular CD4 T cells were significantly negatively correlated with Arg2, H_2_-Q6, and Nfkbia (|cor| > 0.8 and *P* < 0.05) (Supplementary Figure 4). These results suggested that the antinociceptive effect of TET may be related to follicular CD4 T cells.

### 3.7 Molecular docking results

**FIGURE 10:**
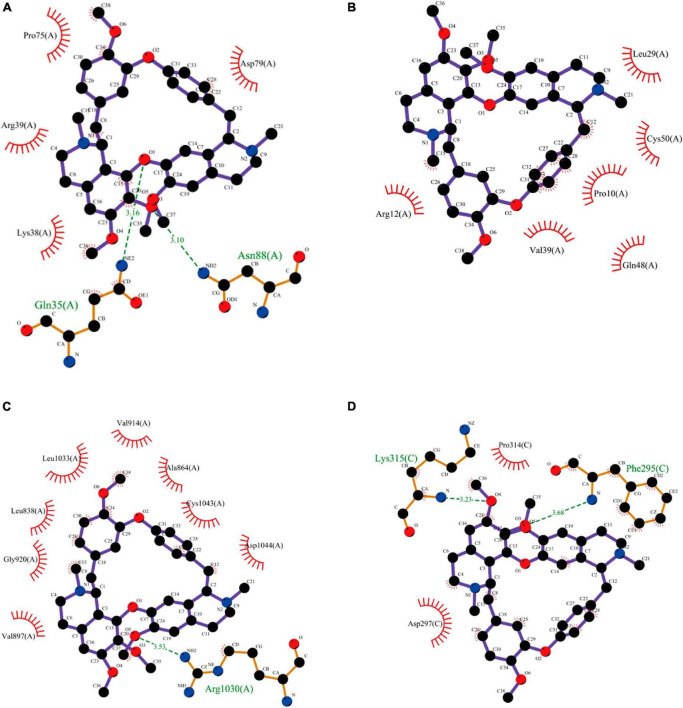
Molecular docking results of TET with Arg2 **(A)**, Cxcl12 **(B)**, Kdr **(C)**, and Nfkbia **(D)**.

To detect the binding energy of proteins encoded by Arg2, Cxcl12, Kdr, and Nfkbia with TET, we performed molecular docking. As results in Figures 10A–D and Table 1 indicate, the binding energy of TET with Arg2, Cxcl12, Kdr, and Nfkbia was less than −7.0 kcal/mol, indicating good binding affinity.

**TABLE 1:** AutoDock docking results of TET with Arg2, Cxcl12, Kdr, and Nfkbia.

Target	Binding energy (kcal/mol)	H-bond interactions	Bond distance (Å)	Hydrophobic interactions
Arg2	−8.3	Asn88	3.10Å	Asp79, Pro75, Arg39, Lys38
		Gln35	3.16Å	
Cxcl12	−7			Arg12, Val39, Gln48, Pro10, Cys50, Leu29
Kdr	−8.8	Arg1030	3.53Å	Asp1044, Cys1043, Ala864, Val914, Leu1033, Leu838, Gly920, Val897
Nfkbia	−7.7	Phe295	3.68Å	Pro314, Asp297
		Lys315	3.23Å	

## 4 Discussion

This study is the first to uncover the anti-CINP effects of TET. TET alleviated mechanical allodynia in oxaliplatin injected mice. Arg2, Cxcl12, Kdr, H_2_-Q6, and Nfkbia were identified as core inflammation-related molecules in TET’s antinociceptive action. Among these, the qPCR results suggest that Cxcl12 is an important molecule in TET’s antagonism of OINP, while molecular docking results indicate that Kdr is the most likely potential target through which TET exerts its effects.

Oxaliplatin, a widely used chemotherapy drug known to cause peripheral neuropathy, was employed to induce mechanical allodynia in mice. In this study, we discovered that both TET and pregabalin significantly reduced the mechanical allodynia triggered by oxaliplatin. Notably, the dosages of TET and pregabalin (45 mg/kg) adhered to the recommended dose based on human/mouse dosage conversion (Nair et al., 2018), as indicated in the instructions. It was observed that while 45 mg/kg of TET did not impair the mice’s mobility or induce drowsiness, pregabalin did cause drowsiness (Zhang et al., 2023). Complementing these findings, our prior research also indicated that TET alleviated NP resulting from nerve damage (Zhang et al., 2023). Collectively, these results suggest that TET is a promising candidate for OINP therapy.

Cytokines, chemokines, and their receptors play important roles in mediating the activation of glial cells and neurons, the production of cytokines, and infiltration of immune cells, which promote the pathogenesis of chronic pain (Koper et al., 2018; Liu et al., 2019; Vergne-Salle and Bertin, 2021). In this study, DEGs from both Control-Oxaliplatin and Oxaliplatin-TET groups were highly enriched in inflammation processes including the negative regulation of cytokine production, chemokine receptor binding, antioxidant activity, chemokine activity, CXCR chemokine receptor binding, chemokine signaling pathway, cytokine-cytokine receptor interaction, and the MAPK signaling pathway. In the context of chronic pain, the interaction between neurons, astrocytes, and microglial cells may be mediated by the activated cytokine and chemokine signaling pathways. Oxaliplatin can induce the production of certain pro-inflammatory cytokines in SC cells, including IL-18, TNF-α, and MMP2 (Huang et al., 2021; Bai et al., 2023). These cytokines can bind to their respective receptors, enhancing glutamate activity and inhibiting inhibitory synaptic transmission, leading to increased excitability of neurons in the SC and subsequently chronic pain (Liu et al., 2017; Zhang et al., 2017). The mitogen-activated protein kinase (MAPK) signaling pathway, associated with inflammation induction and the maintenance of OINP, includes the activation of p38, extracellular signal-regulated kinase (ERK), and c-Jun N-terminal kinase (JNK) (Abd-Elmawla et al., 2023). Our findings suggest that targeting the cytokine-receptor interaction, chemokine signaling, or the MAPK pathway could be effective strategies for treating OINP. This suggests that TET might exert its anti-OINP effects by modulating the chemokine pathway.

Increased expression of CXCL12 has been observed in the dorsal root ganglia of mice with OINP. Intrathecal administration of anti-CXCL12 neutralizing antibodies or CXCL12 siRNA has been shown to alleviate both mechanical and thermal pain induced by oxaliplatin (Li et al., 2017). Complementary to these findings, our RNA-Seq and qPCR validation results demonstrated an upregulation of Cxcl12 gene expression in the spinal dorsal horn of OINP mice. Furthermore, TET not only counteracted the OINP-induced mechanical allodynia but also reduced Cxcl12 gene expression. These results collectively underscore the critical role of Cxcl12 in the pathogenesis of OINP and highlight the therapeutic potential of targeting this pathway.

Arg2 is an enzyme critical in L-arginine metabolism. In the absence of Arg2 (Arg2^–/–^ mice), there is an observed increase in microglial cell activation and pain behavior following nerve injury (Yin et al., 2020). Despite this, both RNA-Seq and qPCR did not identify any change of Arg2 in this study. However, considering that Arg2 is a metabolic enzyme, it might undergo post-transcriptional modifications, such as phosphorylation, which could impact its activity and role in the progression of OINP. Similarly, Nfibia, known for its ability to inhibit the activation of NF-κB—a crucial factor in neuroinflammation and neuropathic pain (Tóbon-Velasco et al., 2014)—did not show changes in gene expression in our RNA-Seq study. Yet, our qPCR results indicated that treatment with TET significantly upregulated Nfibia expression. Therefore, it seems that both Nfibia and Arg2 could be acting as protective genes. TET may contribute to alleviating OINP by enhancing the functions of both Nfibia and Arg2, despite the absence of detectable changes in their mRNA levels, underscoring the importance of looking beyond gene expression to understand their full roles in disease progression and treatment.

Kdr, also known as VEGFR-2 (vascular endothelial growth factor receptor-2), belongs to the class IV receptor tyrosine kinase family (Apte et al., 2019). Increased expression of VEGFR2 was observed in the DRG of CCI mice (Lin et al., 2010). Central vascular endothelial cells expressing VEGFR2 can facilitate the migration of peripheral CD11b + circulating cells to the central nervous system, promoting the development of chronic pain in inflammatory arthritis (Beazley-Long et al., 2018). Although our qPCR analyses did not reveal alterations in VEGFR-2 at the mRNA level, molecular docking studies demonstrated a robust binding affinity between VEGFR-2 and TET. Considering VEGFR-2’s role as a receptor molecule, we propose that TET might mediate its analgesic effects through the association with VEGFR-2, thus obstructing the interaction between VEGFR-2 and its ligands.

The inflammatory response mechanism and immune microenvironment imbalance of CINP are receiving increasing attention from researchers (Lees et al., 2017; Starobova et al., 2020). We found that the infiltration of resting NK cells and plasma cells decreased, while follicular CD4 T cells increased in the Oxaliplatin group, indicating changes in the immune microenvironment in OINP. Since antibodies produced by plasma cells were related to the suppression of neuroinflammation (Wang et al., 2021a,b), the reduction in plasma cells may cause excessive neuroinflammation in NP to be uncontrolled. CD4 T cells are helper cells among T cells (Ruterbusch et al., 2020), and follicular T cells include follicular helper T cells (Tfh) and follicular regulatory T cells (Tfr). Tfr cells inhibit Tfh-mediated antibody responses (Clement et al., 2022). Our results showed that compared to the control, the infiltration of follicular CD4 T cells in the Oxaliplatin group increased, indicating that the increased CD4 T cells here were mainly Tfr, as these Tfr downregulated by TET. This suggested that TET may participate in the immune regulation process by inhibiting the number of Tfr cells.

In summary, this study elucidated the antinociceptive effects of tetrandrine (TET) on oxaliplatin-induced mechanical allodynia in mice, providing new insights into TET’s role in OINP. While the study suggests that inflammatory factors like Cxcl12 play a significant role in TET’s antagonism of OINP, additional interventions are necessary to validate these candidate mechanisms. Efforts should focus on identifying potential targets based on changes in protein expression, and further pharmacological and clinical research is required to fully understand TET’s anti-OINP effects. Additionally, peripheral sensitization is crucial to oxaliplatin-induced neuropathic pain, yet we did not detect changes of Arg2, Cxcl12, Kdr, and Nfkbia in the DRG (data not shown), indicating that the peripheral mechanisms warrant further investigation. Given that inflammation is highly sex-dependent, the anti-OINP effect of TET should also be explored in female mice.

## Data availability statement

The data presented in the study are deposited in the BioProject database repository, accession number PRJNA1049131.

## Ethics statement

The animal study was approved by the Peking University Animal Use Committee. The study was conducted in accordance with the local legislation and institutional requirements.

## Author contributions

Z-LZ: Conceptualization, Data curation, Methodology, Visualization, Writing – original draft, Writing – review and editing, Z-YW: Validation, Writing – review and editing. F-YL: Methodology, Validation, Writing – review and editing. H-YC: Methodology, Validation, Writing – review and editing. S-DZ: Supervision, Writing – review and editing.

## Footnotes

1
https://ftp.ncbi.nih.gov/blast/db/FASTA/

2
http://pfam-legacy.xfam.org/

3
https://www.ncbi.nlm.nih.gov/research/cog/

4
https://www.uniprot.org/help/downloads

5
https://ftp.ncbi.nih.gov/pub/COG/KOG/

6
https://www.genome.jp/kegg/

7
https://www.geneontology.org/

8
http://www.gsea-msigdb.org/gsea/msigdb

9
https://pubchem.ncbi.nlm.nih.gov/

10
https://www.rcsb.org/

11
https://autodocksuite.scripps.edu/adt

12
https://altair.sci.hokudai.ac.jp/g6/service/pocasa/
