# Supplementary material for: Tetrandrine alleviates oxaliplatin-induced mechanical allodynia via modulation of inflammation-related genes
Source: Front Mol Neurosci. 2024 Feb 14;17:1333842. doi: 10.3389/fnmol.2024.1333842 (PMC10899404; doi:10.3389/fnmol.2024.1333842)
Supplement: Supplementary file 9 [file Data_Sheet_1.doc]

**Supplementary Materials and methods**

**Cold Allodynia**

This specific experiment utilized a hot/cold plate apparatus (Bioseb, France). The temperature of the cold plate was maintained at 2.5 ℃ to measure cold allodynia. During this test, animals were placed on a 16 × 16 cm cold plate enclosed within a 25 cm high Plexiglass cylinder to determine baseline latencies for pain reactions. These reactions included lifting, biting, shaking of hind paws, jumping, movement deficits, or writhing responses. To prevent potential damage to paw tissue, a cutoff time of 60 seconds was established.

**Supplementary Figures**

**Figure S.1** Effects of oxaliplatin on cold allodynia in the cold plate. The latency to pain reaction was examined -1, 1, 4, and 7 days after Vehicle (5% glucose solution) or Oxaliplatin injection (i.p.), Data are presented as mean ± SEM, n = 6.


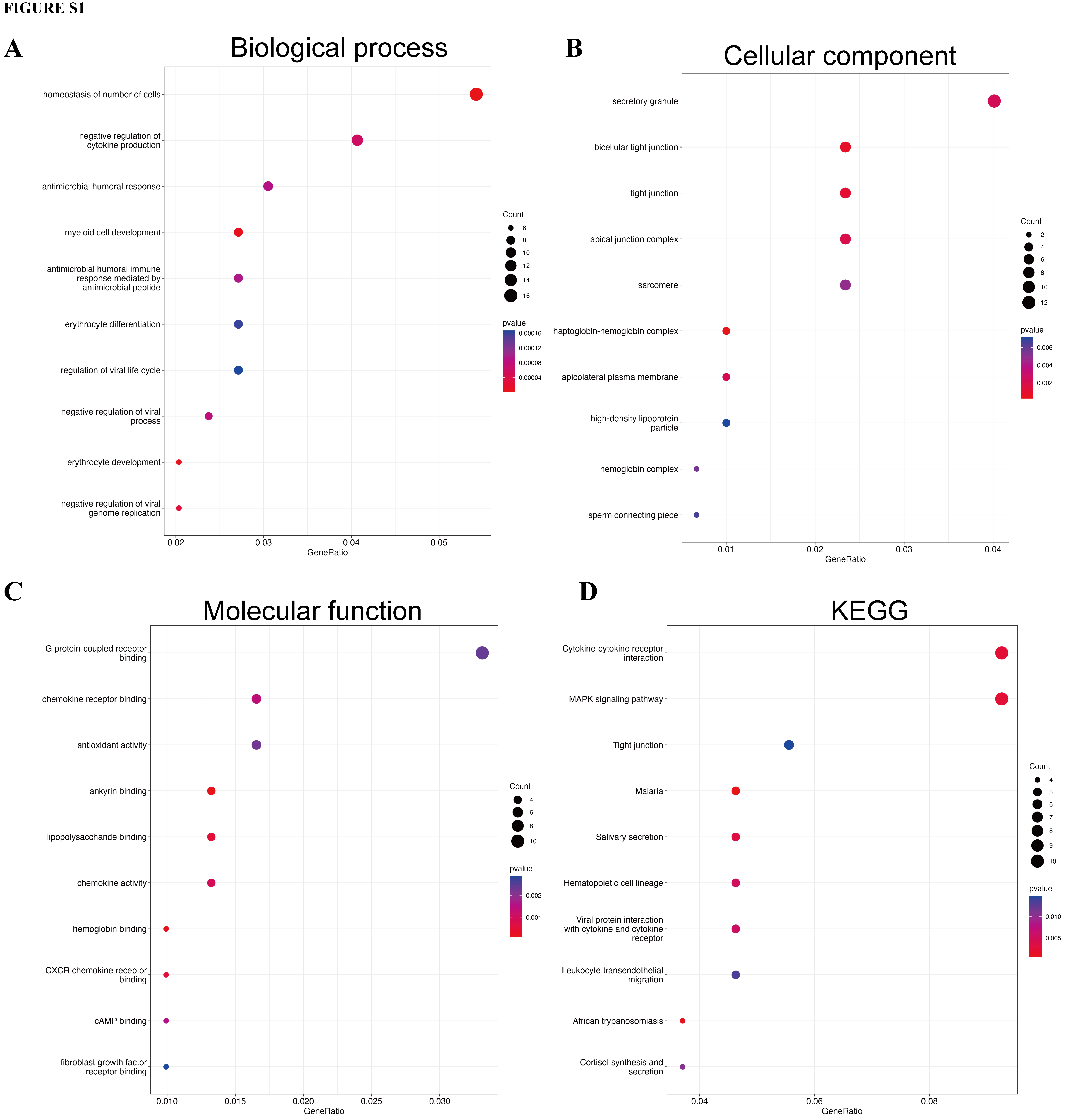


**Figure S.2** Functional enrichment analysis of DEGs in Control vs Oxaliplatin groups using clusterProfiler. The bubble charts showed BP (A), CC (B), MF (C), and KEGG (D).


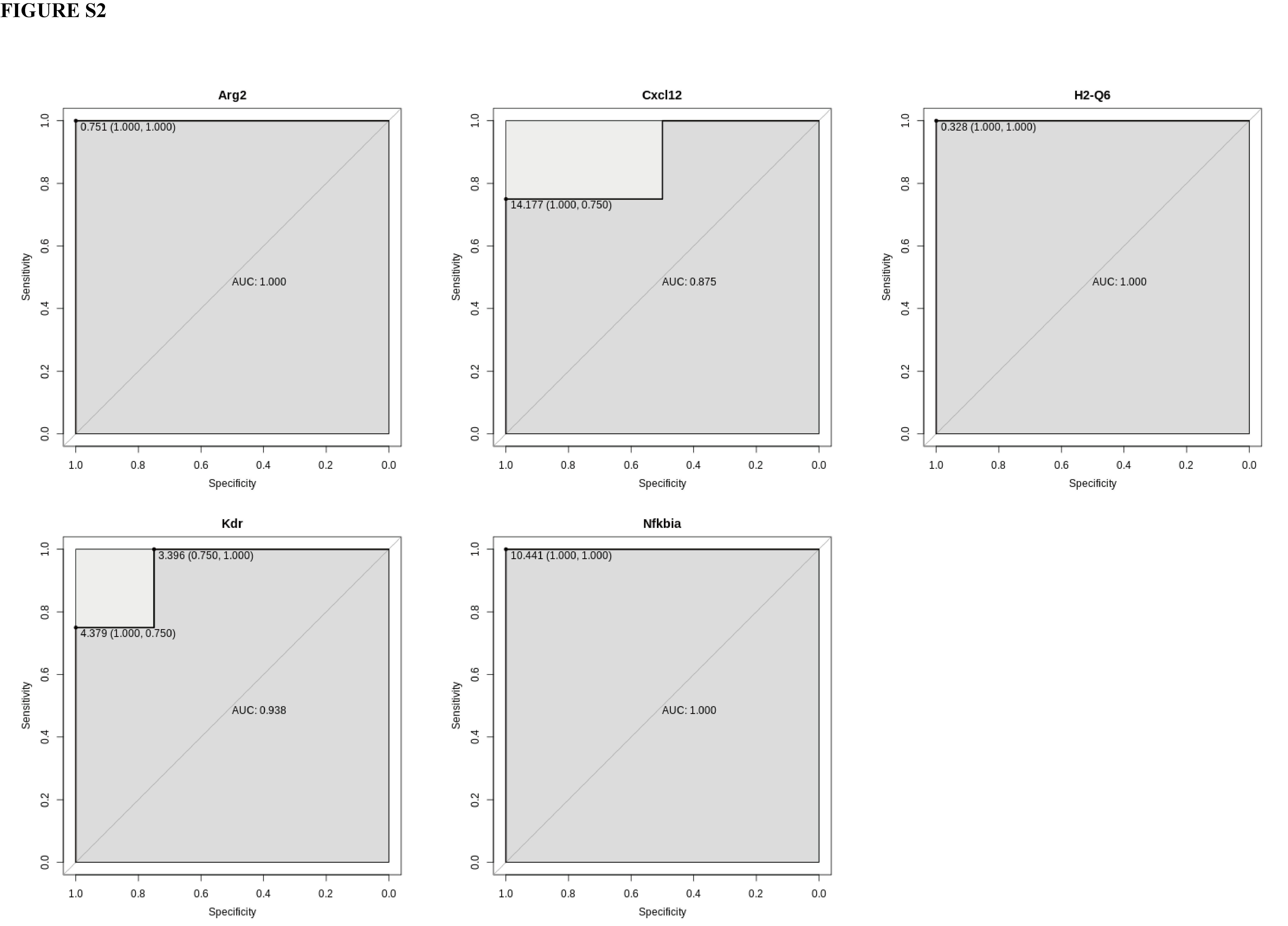


**Figure S.3** ROC analysis of Arg2, Cxcl12, Kdr, H2-Q6, and Nfkbia in the Oxaliplatin-TET group.


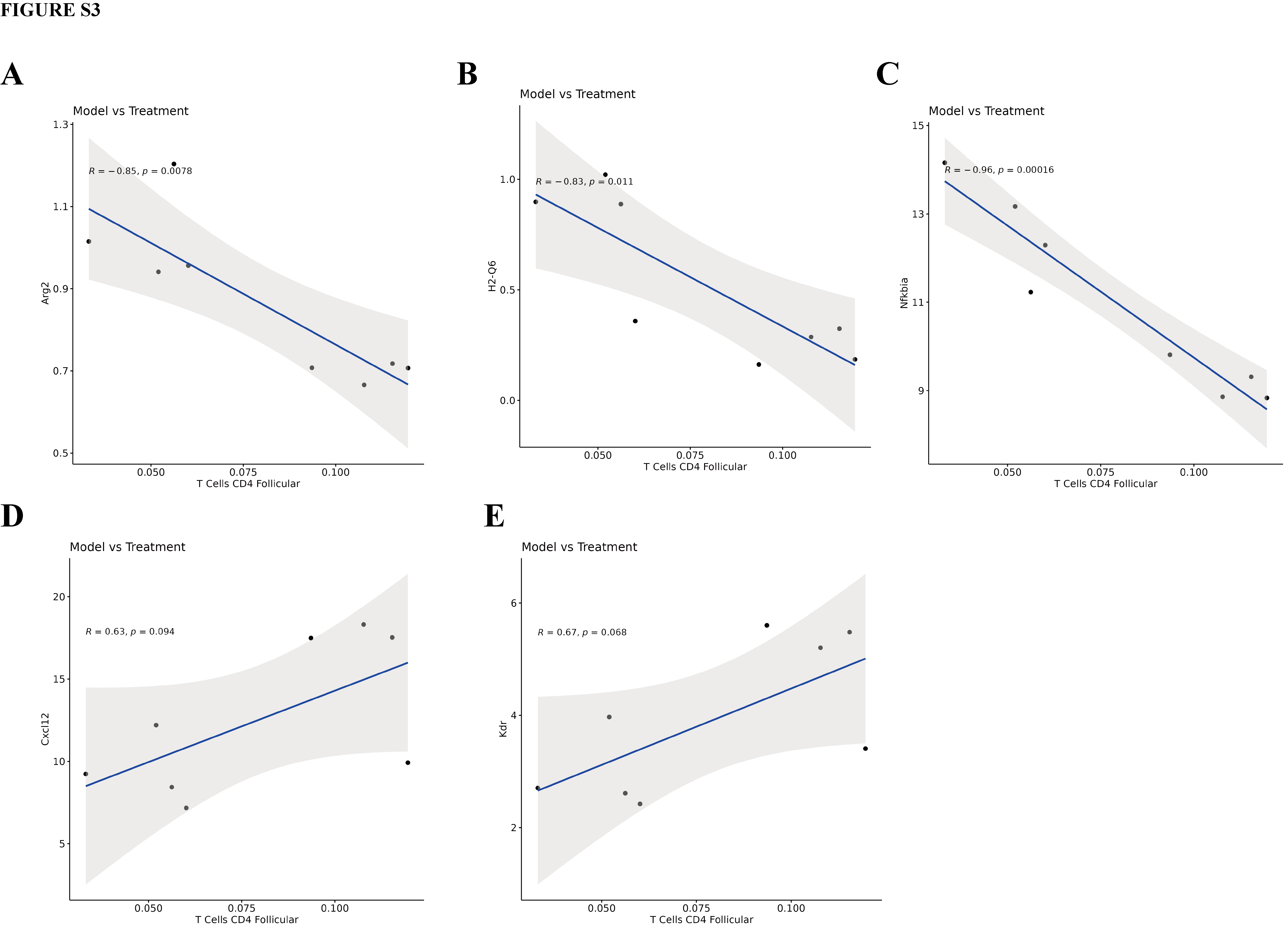


**Figure S.4** Correlation analyzes between T cells CD4 follicular and Arg2 (A), H2-Q6 (B), Nfkbia (B), Cxcl12 (C), or Kdr (C).
